# Intraoperative hypotension and postoperative acute kidney injury or myocardial injury after liver resection: A protocol for a systematic review and meta-analysis

**DOI:** 10.1371/journal.pone.0357750

**Published:** 2026-10-12

**Authors:** Yi Duan, Meng Li, Mengzhuo Guo, Shengda Li, Zhifeng Gao

**Affiliations:** Department of Anesthesiology, Beijing Tsinghua Changgung Hospital, School of Clinical Medicine, Tsinghua Medicine, Tsinghua University, Beijing, China; Cleveland Clinic, UNITED STATES OF AMERICA

## Abstract

**Background:**

Liver resection creates a clinical tension between limiting operative bleeding and maintaining organ perfusion. Low central venous pressure, fluid restriction, vascular inflow occlusion and vasoactive treatment can coincide with lower arterial pressure, yet existing reviews have centred on blood loss and transfusion. They have not resolved whether directly quantified intraoperative hypotension is associated with postoperative acute kidney injury or myocardial injury, or whether comparative haemodynamic strategies alter these outcomes. We will address both questions while keeping exposure associations distinct from management effects and kidney outcomes distinct from myocardial outcomes.

**Methods:**

This prospectively registered review will search four bibliographic databases and two trial registries from inception, supplemented by citation searching and author contact, without language or date restrictions. Eligible studies will include adults undergoing partial liver resection and report a quantified intraoperative arterial-hypotension exposure or a comparative pressure- or flow-directed strategy with acute kidney injury or myocardial injury. Two reviewers will independently select studies, extract data and assess risk of bias, with independent handling of review-team-authored studies. Four prespecified synthesis domains will separate exposure from management evidence and kidney from myocardial outcomes. Compatible estimates will undergo random-effects meta-analysis; otherwise, structured synthesis without meta-analysis will be used. Heterogeneity, missing evidence and certainty will be assessed. This design aims to distinguish prognostic association from evidence about modifiable haemodynamic management.

**Registration:**

PROSPERO CRD420261476933; Open Science Framework https://doi.org/10.17605/OSF.IO/3GCY2.

## Introduction

During partial liver resection, anaesthetic and surgical teams may use low central venous pressure, fluid restriction, vascular inflow occlusion and vasoactive drugs to reduce venous congestion and operative bleeding. Systematic reviews of strategies targeting low central venous pressure suggest reductions in blood loss and transfusion, but definitions and cointerventions vary, as does the certainty of the evidence [1–3]. Central venous pressure, however, does not directly reflect arterial pressure or organ perfusion. A low venous-pressure strategy may be implemented while arterial pressure is preserved, whereas arterial hypotension may result from blood loss, anaesthetic vasodilation, inflow occlusion or deliberately restrictive haemodynamic management.

Across non-cardiac surgery, lower intraoperative arterial pressure, longer duration below specified pressure thresholds and greater area under those thresholds have been associated with adverse postoperative outcomes [4]. Acute kidney injury (AKI) after surgery is associated with postoperative morbidity and may be underdetected when postoperative renal surveillance is incomplete [5]. Myocardial injury after non-cardiac surgery is often detectable only through systematic perioperative troponin testing and may occur without ischaemic symptoms [6]. Both outcomes are therefore susceptible to differences in ascertainment across studies of liver resection.

Existing reviews of liver resection have centred on low central venous pressure, operative blood loss and transfusion rather than quantified intraoperative arterial hypotension burden and postoperative kidney or myocardial injury [1–3]. Treating distinct forms of haemodynamic evidence as equivalent could lead an exposure-outcome association to be misinterpreted as evidence that raising arterial pressure improves outcomes. This review will therefore analyse direct exposure evidence and comparative management evidence separately.

This review will determine, among adults undergoing partial liver resection, (1) the association between directly quantified intraoperative arterial hypotension burden and postoperative AKI, (2) the association between the same exposure and postoperative myocardial injury, (3) the effects of comparative intraoperative pressure- or flow-directed management strategies on AKI, and (4) the effects of these strategies on myocardial injury. We will also investigate whether results vary according to exposure and outcome definitions, surgical characteristics and surveillance intensity, and will assess the certainty and applicability of the evidence and identify evidence gaps.

## Methods

### Design, reporting and registration

This protocol was developed in accordance with the Preferred Reporting Items for Systematic Review and Meta-Analysis Protocols (PRISMA-P) 2015 statement and its explanation and elaboration [7–9]. The completed PRISMA-P checklist accompanies this manuscript as S1 Checklist.

The review is prospectively registered in PROSPERO (CRD420261476933) and on the Open Science Framework (https://doi.org/10.17605/OSF.IO/3GCY2) [10]. Protocol version 1.0 is dated 13 August 2026. The final review will be reported using PRISMA 2020 and PRISMA-S [11,12].

The protocol was developed in August 2026. At this revision, the definitive searches, duplicate screening, data extraction, risk-of-bias assessment and synthesis have not begun. Participant recruitment is not applicable to this systematic review. Definitive searches are planned for 28 September 2026. Data collection, including study screening and data extraction, is expected to be completed by 20 October 2026, and the review results are expected by 10 February 2027.

### Use of artificial intelligence tools and technologies

The authors used OpenAI Codex (OpenAI; accessed 14–17 August 2026) for English-language editing and cross-document consistency checks. The tool was not used to originate the research question, eligibility criteria, outcome definitions, risk-of-bias framework or statistical methods. Substantive edits were limited to alignment with the publicly registered protocol and PLOS ONE requirements. All AI-assisted edits were checked against the registration and cited sources, and the authors take full responsibility for the final content.

### Review questions and analytic domains

The review question is: in adults undergoing partial liver resection, what is the association between directly measured intraoperative arterial-hypotension burden and postoperative AKI or myocardial injury, and what are the effects of comparative intraoperative pressure- or flow-directed strategies on these outcomes? Table 1 summarises the frameworks for population, exposure, comparator and outcome and for population, intervention, comparator and outcome.

**Table 1:** Frameworks for population, exposure, comparator and outcome and for population, intervention, comparator and outcome.

Element	Definition
Population	Adults undergoing open, laparoscopic or robotic partial liver resection, including living-donor hepatectomy.
Exposure	Directly measured intraoperative arterial hypotension, expressed by threshold, relative decrease, duration, area, time-weighted average, episodes, dose category or nadir.
Intervention	A comparative intraoperative pressure-targeted, flow-directed or multicomponent strategy with an explicit haemodynamic target, treatment trigger or adjustment algorithm.
Comparator	Lower hypotension burden or reference category; usual care; or another haemodynamic target or strategy.
Co-primary outcomes	Postoperative AKI and postoperative myocardial injury, analysed separately.
Eligible designs	Randomised and non-randomised comparative intervention studies, cohort studies, controlled before-after studies and eligible case-control studies.

Abbreviation: AKI, acute kidney injury.

### Eligibility criteria

Table 2 presents the eligibility criteria.

**Table 2:** Eligibility criteria.

Domain	Include	Exclude
Participants	Adults aged 18 years or older undergoing elective or emergency partial liver resection for malignant or benign disease or living donation; a study’s adult threshold will be accepted if broadly equivalent.	Liver transplantation; paediatric-only cohorts; ablation-only or diagnostic procedures; non-hepatic surgery; mixed cohorts without extractable or author-obtainable liver-resection data.
Exposure/strategy	Directly quantified intraoperative MAP or systolic-pressure hypotension from invasive or non-invasive measurements, or a comparative pressure-/flow-directed strategy with an explicit target or treatment algorithm and eligible outcomes.	Purely postoperative hypotension; monitoring alone; low CVP, fluid restriction, transfusion, epidural, lactate or near-infrared spectroscopy alone without an eligible arterial-pressure exposure or comparative management algorithm.
Outcomes	AKI within 7 postoperative days (or during the index admission when day 7 is unavailable) and/or myocardial injury within 30 days; prespecified secondary renal, cardiac, mortality and complication outcomes.	No eligible kidney or myocardial outcome; outcome data not separable from an ineligible population or time window.
Study design	Randomised or cluster-randomised trials, non-randomised comparative intervention studies, cohort studies, controlled before-after studies and eligible case-control studies.	Case reports or case series, uncontrolled intervention studies, reviews, editorials, protocols without results, simulations, animal or in-vitro studies.
Report characteristics	Any language, year, country or publication status; conference reports, dissertations and registry results if eligibility, an effect estimate and risk of bias can be assessed.	Retracted reports; records with insufficient information after reasonable author contact; duplicate reports that add no unique data.

Abbreviations: MAP, mean arterial pressure; CVP, central venous pressure; AKI, acute kidney injury.

### Participants and setting

We will include adults undergoing open, laparoscopic or robotic partial liver resection for malignant or benign disease and healthy living donors. Major and minor resections, elective and emergency procedures and studies using a broadly equivalent adult threshold will be eligible. We will exclude transplantation, paediatric-only surgery, ablation-only or diagnostic procedures and non-hepatic surgery. Mixed surgical cohorts will be included only when liver-resection data are separately extractable or can be obtained from study authors.

### Exposure and intervention definitions

Eligible exposure studies must quantify intraoperative arterial hypotension from invasive or non-invasive measurements. Eligible metrics include absolute mean or systolic arterial-pressure thresholds, relative decreases from baseline, minutes below a threshold, time-weighted average, area or cumulative exposure below a threshold, number or duration of episodes, dose categories and nadir pressure. Combined perioperative exposure will be accepted only if the intraoperative component is separable or demonstrably predominant; inseparable exposures will be tested in sensitivity analysis. Purely postoperative exposure will be excluded.

Eligible comparative management studies will evaluate an intraoperative strategy with an explicit arterial-pressure or flow-related target, treatment trigger or adjustment algorithm. Eligible approaches include alternative arterial-pressure targets, vasoactive titration protocols and fluid or drug adjustment guided by cardiac output, cardiac index or stroke-volume measures. Monitoring without a corresponding treatment algorithm is insufficient. Strategies will be classified as pressure-targeted, flow-directed or multicomponent according to their targets and treatment components. Eligibility will be based on the planned strategy rather than achieved pressure differences or outcome effects.

A low-central-venous-pressure or fluid-restriction protocol, transfusion strategy, epidural regimen, lactate target or near-infrared-spectroscopy strategy alone will not qualify without an eligible haemodynamic target or treatment algorithm. Studies of these approaches reporting a quantified arterial-hypotension association may contribute to the exposure domains. Effects of multicomponent protocols will be attributed to the whole strategy unless a pressure-specific effect can be isolated by design.

For exposure studies, comparators may be a lower exposure group, an explicit reference category, a higher pressure category or a continuous contrast. For management studies, comparators may be usual care, another pressure target or another eligible haemodynamic strategy.

### Outcomes

The co-primary outcomes will be analysed separately. AKI will be assessed within 7 postoperative days, or over the index admission if a day-7 estimate is unavailable. Kidney Disease: Improving Global Outcomes criteria will be preferred. AKIN, RIFLE and study-specific definitions will be retained, mapped where defensible and examined separately. When several eligible AKI time points are reported, the estimate closest to postoperative day 7 will be primary.

Myocardial injury will be defined primarily as postoperative cardiac troponin above the assay-specific 99th-percentile upper reference limit within 30 days. Myocardial injury after non-cardiac surgery (MINS) will be retained as a distinct definition when ischaemic adjudication or an explicit MINS algorithm is reported, with the handling of non-ischaemic causes recorded. Symptoms or electrocardiographic changes will not be required for the broader myocardial-injury outcome. When multiple eligible time points are reported, the estimate closest to 30 days will be primary. AKI and myocardial injury will never be combined into one pooled organ-injury endpoint.

We will classify myocardial-injury results by the information available to establish acuity: (1) preoperative and postoperative measurements supporting a new elevation or acute change, (2) no preoperative measurement but serial postoperative measurements supporting an acute rise or fall, or (3) postoperative elevation of uncertain acuity because the available measurements do not distinguish acute from chronic injury. Study-specific change criteria will be recorded. Results without a preoperative value remain eligible, but postoperative dynamics alone do not establish the timing of injury onset relative to surgery.

The broad troponin-defined outcome may include results of uncertain acuity, which will be identified and examined separately from results supporting acute injury. Stable pre-existing elevations will not be classified as incident acute injury. Where aggregate data do not distinguish acute injury from stable pre-existing elevations, the study definition will be retained for the broad outcome and this limitation recorded. These results will be excluded from sensitivity analyses limited to acute injury supported by troponin measurements. Reported new ischaemic electrocardiographic changes, new regional wall-motion abnormalities and cardiac adjudication will be extracted as supporting information. These findings will neither replace troponin nor be required for eligibility for the broad troponin-defined outcome [6].

Secondary outcomes will include moderate or severe AKI, renal replacement therapy, myocardial infarction, in-hospital or 30-day mortality and reported complications or adverse effects of haemodynamic management. Composite outcomes will be extracted, but component-specific results will be prioritised.

### Study designs and report characteristics

Eligible designs will include individual or cluster randomised trials, non-randomised comparative intervention studies, prospective or retrospective cohort studies, controlled before-after studies and case-control studies. In case-control studies, cases must be defined by AKI or myocardial injury and controls must arise from the same source population without selection on post-exposure consequences.

We will exclude case reports, case series, uncontrolled intervention studies, reviews, editorials, protocols without results, simulations, animal studies and in-vitro studies. No language, year, country or publication-status restrictions will be applied. Conference reports, dissertations and registry results will be eligible only if the population, exposure or intervention, outcome, effect estimate and risk of bias can be assessed. Retracted reports will be excluded; corrections and expressions of concern will be evaluated before use.

### Information sources and search strategy

We will search PubMed, Embase.com, Web of Science Core Collection and CENTRAL from inception. ClinicalTrials.gov and the WHO International Clinical Trials Registry Platform will be searched without an outcome restriction. We will also examine reference lists of included studies and relevant reviews, conduct forward citation and PubMed related-article searches and contact investigators when clarification or unreported eligible data are required. Scite will be consulted for citation context and notices of correction or retraction.

Yi Duan developed the draft PubMed strategy. Before the definitive search, Meng Li, who did not participate in its development, will independently peer-review it using the PRESS 2015 evidence-based checklist [13]. Yi Duan will address the review comments before Meng Li translates the approved strategy for Embase.com, Web of Science Core Collection and CENTRAL. The search combines two paths: liver resection with arterial-pressure or haemodynamic concepts, and liver resection combined with kidney or myocardial injury concepts. No language, date, adult or study-design filters will be applied electronically. The draft PubMed strategy and database-translation methods are provided in S1 File.

For each source, we will report the database or register, platform, complete strategy, search date, coverage and number of records before deduplication. Changes made during database translation will be documented. If final analysis occurs more than 6 months after the initial search, all bibliographic searches will be updated.

### Study records and selection

Search results will be exported to reference-management software and then to review software after automated and manual deduplication. Record counts from each source and each deduplication step will be reported. Machine-learning prioritisation may be used only to order records for screening; no record will be excluded without human assessment.

Yi Duan and Mengzhuo Guo will independently screen titles and abstracts and then full texts. They will pilot 50 title and abstract records to calibrate application of the eligibility criteria. Any change to those criteria will be documented as a protocol amendment. Disagreements will be resolved by discussion and then by Zhifeng Gao. Full-text exclusions will be recorded with one primary reason, and selection will be reported in a PRISMA flow diagram.

For any report authored by Yi Duan or Zhifeng Gao, neither author will participate in eligibility decisions. Mengzhuo Guo and Meng Li will screen independently, with Shengda Li adjudicating unresolved disagreements.

### Data collection and management

A structured extraction form and data dictionary will be piloted on three to five eligible studies. Yi Duan and Mengzhuo Guo will independently extract data, compare entries and resolve discrepancies through discussion or adjudication by Zhifeng Gao. For review-team-authored studies, Mengzhuo Guo and Meng Li will extract independently and Shengda Li will adjudicate.

Investigators will be contacted when eligibility, subgroup data, exposure definitions, denominators, outcome methods, overlap or effect estimates cannot be resolved from the report. One reminder will be sent after 14 days. Queries will normally close after 28 days unless an investigator indicates that a response is imminent.

### Data items and multiplicity

We will extract publication and study identifiers; country, centre and recruitment period; design; eligibility criteria; sample size; age; sex; baseline renal function; chronic kidney disease; cardiovascular disease; cirrhosis, liver function and indication for resection; open versus minimally invasive approach; major versus minor resection; living-donor status; vascular inflow occlusion; blood loss, transfusion, fluid and vasoactive management; arterial-pressure measurement method and interval; exposure threshold, baseline, metric and units; intervention and comparator details; outcome definitions, surveillance schedules and follow-up; numbers analysed; missing data; effect estimates with precision; adjustment variables; funding and conflicts.

For comparative strategies, we will record target values, monitoring variables, treatment triggers, drugs, fluids, rescue measures, cointerventions and comparator care. For myocardial outcomes, we will record the troponin assay and upper reference limit, baseline sampling, postoperative schedule, serial-change criteria, routine versus symptom-triggered testing, and reported electrocardiographic, echocardiographic and adjudication findings.

For multiple exposure specifications, we will first select the authors’ prespecified or primary exposure. If several specifications are equally eligible, cumulative measures incorporating depth and duration will be preferred, followed by duration below a threshold, binary occurrence and nadir. Within the selected exposure definition, model selection will follow the confounding criteria below. Selection will not depend on effect magnitude or statistical significance, and all eligible specifications will be recorded. Correlated estimates from the same cohort will not be treated as independent. For multiple outcome definitions, KDIGO AKI and the broad 99th-percentile troponin definition will be primary, with MINS and alternative definitions retained separately.

Potentially overlapping cohorts will be mapped by centre, recruitment dates, sample size and participant characteristics. The most complete, least biased report will provide the primary estimate for a domain; unique outcomes or time points from companion reports may be used without double counting participants.

### Confounding and model selection

Prespecified confounding domains are age, sex, baseline arterial pressure and hypertension, diabetes, preoperative renal function, cardiovascular disease, liver disease severity, indication for resection, planned resection extent, operative approach and urgency. For AKI, baseline creatinine, estimated glomerular filtration rate or chronic kidney disease will be central to assessment. For myocardial outcomes, prior coronary disease or heart failure and baseline renal function will be central, with baseline troponin recorded when available. We will record how each study addressed these confounding domains through restriction, matching, stratification or statistical adjustment.

Blood loss, transfusion, vasopressor use and fluid administration will be extracted with their timing relative to hypotension and their inclusion in each model. Their potential roles as confounders, mediators or post-exposure treatments will be assessed in light of their timing and clinical context. In studies of repeated hypotension, a variable affected by an earlier episode may also act as a confounder for a later episode. We will flag cumulative intraoperative totals when their timing relative to hypotension cannot be determined. We will record any reported methods used to address time-varying confounding.

Within each cohort, for a selected exposure definition and outcome, we will prefer estimates that address relevant pre-exposure confounding domains and avoid inappropriate adjustment for downstream mediators or treatment responses. For studies of repeated hypotension, we will also consider whether the analysis appropriately addresses time-varying confounding. If equally suitable models remain, we will prefer the authors’ prespecified primary model, where identified. Estimates with limited confounding control, unclear treatment timing or potentially inappropriate adjustment for downstream variables will be flagged for risk-of-bias assessment and sensitivity analysis. We will synthesise reported adjusted estimates and record the covariate-based rationale for model selection.

### Risk-of-bias assessment

Two reviewers will independently assess risk of bias at the result level. Randomised trials will be assessed with RoB 2 (22 August 2019 version) [14]. Non-randomised intervention studies will be assessed with the final 2016 ROBINS-I tool [15]. Observational follow-up studies estimating exposure effects will be assessed with ROBINS-E (24 March 2024 version) [16]. Eligible case-control studies not adequately covered by ROBINS-E will receive item-level Joanna Briggs Institute case-control appraisal without a numerical summary score.

ROBINS-E overall judgements will follow its domain algorithms and overall guidance. The highest domain rating will generally determine the overall rating. We will justify any departure, including a higher overall risk-of-bias rating when several domains raise concerns. The four overall levels are Low risk of bias, Some concerns, High risk of bias and Very high risk of bias. Predicted bias direction and threats to conclusions will be recorded separately.

Yi Duan and Mengzhuo Guo will perform independent assessments, with Zhifeng Gao adjudicating. For review-team-authored reports, Mengzhuo Guo and Meng Li will assess independently and Shengda Li will adjudicate. Domain judgements and supporting rationale will be reported.

### Effect measures

For dichotomous intervention outcomes, risk ratios with 95% confidence intervals will be preferred when available. For randomised comparisons, the primary estimand will be the intention-to-treat effect of assignment; per-protocol effects will be secondary when reported. For observational exposure studies, adjusted risk ratios, odds ratios and hazard ratios will be extracted and synthesised on separate effect-measure scales unless a defensible conversion is prespecified. Continuous exposures will be harmonised to clinically interpretable units only when the exposure contrast and functional form are compatible. Ordinal exposure categories will be retained when linearity cannot be assumed.

### Sample size and analysis software

No fixed sample size is specified because all studies meeting the prespecified eligibility criteria will be included. Analyses will be conducted in R (R Foundation for Statistical Computing, Vienna, Austria). The exact R version and package versions used will be recorded in the archived analysis code and reported in the final review.

### Data synthesis

Four primary synthesis domains are prespecified (Table 3). Studies will be pooled only when at least two estimates address a compatible population, exposure or strategy, outcome definition, effect measure and estimand. The presence of two studies alone will not be considered sufficient if clinical or methodological incompatibility is material. Adjusted observational estimates will be prioritised; unadjusted estimates will be synthesised separately or used in sensitivity analysis. Management strategies will be grouped by pressure-targeted, flow-directed or multicomponent approach, with pooling within groups subject to these compatibility criteria.

**Table 3:** Prespecified primary synthesis domains.

Domain	Evidence	Outcome	Primary analytic emphasis
1	Direct hypotension exposure	Postoperative AKI	Adjusted exposure-outcome estimates selected by prespecified confounding criteria; unadjusted estimates analysed separately or in sensitivity analyses.
2	Direct hypotension exposure	Postoperative myocardial injury	Broad troponin-defined injury and MINS kept distinct; baseline and serial measurements used to classify acuity.
3	Comparative management strategy	Postoperative AKI	Pressure-targeted, flow-directed and multicomponent strategies grouped separately; randomised and non-randomised estimates kept distinct unless design-compatible synthesis is justified.
4	Comparative management strategy	Postoperative myocardial injury	Strategy effects interpreted independently of exposure associations; troponin ascertainment groups retained.

Abbreviations: AKI, acute kidney injury; MINS, myocardial injury after non-cardiac surgery.

All eligible studies will remain visible in the descriptive evidence inventory. Non-randomised intervention results at Critical risk of bias using ROBINS-I and exposure results at Very high risk of bias using ROBINS-E will be excluded from the primary effect synthesis and presented separately. ROBINS-E results at Low risk of bias, Some concerns or High risk of bias will remain eligible when other synthesis criteria are met. High-risk results will be identified and removed in sensitivity analysis. These rules apply to individual outcome estimates. The separate ROBINS-E judgement about threats to conclusions will inform interpretation rather than determine eligibility for the primary synthesis.

When pooling is appropriate, we will use random-effects meta-analysis with between-study variance estimated by restricted maximum likelihood and Hartung-Knapp inference. We will report the pooled effect with a 95% confidence interval, tau-squared, I-squared and Cochran’s Q; a 95% prediction interval will be reported when at least five sufficiently similar studies contribute. Cluster-randomised estimates must account for clustering, and shared comparator groups in multi-arm studies will be split or modelled to prevent double counting. Studies with no outcome events in either group will not contribute relative-effect information to conventional pairwise meta-analysis but will be described. Studies with an event in only one group will be handled using a method appropriate to the selected model rather than a universal continuity correction.

No study or effect estimate will be excluded solely because it appears statistically extreme. Extreme estimates will be checked for extraction, unit or denominator errors, overlapping samples, incompatible estimands and clinically or methodologically distinct characteristics. Verified eligible estimates will be retained, with their influence evaluated through heterogeneity measures and sensitivity analyses.

When meta-analysis is not appropriate, findings will be grouped by analytic domain, study design, exposure or intervention definition and outcome and reported using the Synthesis Without Meta-analysis guideline [17]. Direction and magnitude of effects, precision and risk of bias will be displayed in structured tables or plots. We will not use vote counting based only on statistical significance.

### Heterogeneity and additional analyses

Prespecified sources of heterogeneity include open versus minimally invasive surgery; major versus minor resection (major defined as three or more Couinaud segments or the study’s definition); MAP threshold families below 65, 60 and 55 mm Hg; KDIGO versus other AKI definitions; protocolised versus non-protocolised troponin surveillance; availability of preoperative and serial postoperative troponin measurements and the resulting acuity classification; and living donors versus patients with liver disease. Subgroup differences will be tested by formal interaction rather than by comparing within-subgroup significance. Meta-regression or multivariable exploration will be considered only when at least 10 studies per parameter provide compatible data; otherwise analyses will be descriptive and labelled exploratory.

### Sensitivity analyses

Where data permit, sensitivity analyses will exclude High-risk ROBINS-E results and other high-risk results retained in the primary synthesis. For myocardial injury, we will restrict analyses to results with measurements supporting a new elevation or acute change, distinguishing those with a preoperative baseline from those supported only by postoperative dynamics. Results of uncertain acuity, including aggregate results that do not distinguish acute injury from stable pre-existing elevations, will be excluded from these analyses. Separate sensitivity analyses will exclude estimates with inadequate control of relevant prespecified confounding domains and estimates with clearly inappropriate adjustment for downstream mediators or treatment responses. In judging adjustment for downstream variables, we will consider any methods used to address time-varying confounding. Other sensitivity analyses will address non-KDIGO AKI definitions, composite-only outcomes, non-peer-reviewed records, incomplete troponin surveillance, unadjusted estimates, overlapping cohorts, inseparable perioperative exposure and alternative reasonable random-effects specifications. Analyses requiring pooling will be performed only when at least two compatible estimates remain. Sensitivity findings will be interpreted alongside the primary results.

### Reporting biases

We will compare publications with protocols, registrations and statistical analysis plans when available and will seek unreported prespecified outcomes. For eligible pairwise intervention meta-analyses, risk of bias due to missing evidence will be assessed with ROB-ME [18]. Missing-evidence concerns in exposure studies and other syntheses will be evaluated narratively. Funnel plots and asymmetry tests will be considered only when at least 10 sufficiently comparable studies are available and will not be interpreted as specific proof of publication bias.

### Certainty of evidence and interpretation

Certainty will be rated separately for each analytic domain and co-primary outcome. Standard GRADE domains will be applied to comparative management evidence, and adapted prognostic-factor guidance will be applied to exposure evidence [19]. Judgements will consider risk of bias, inconsistency, indirectness, imprecision, publication or missing-evidence bias and any justified upgrading considerations. Evidence that hypotension is associated with injury will not be interpreted as proof that a strategy to raise arterial pressure prevents injury.

Interpretation will consider consistency, temporal and biological plausibility, risk of bias and comparative management evidence. It will also consider ROBINS-E judgements on the predicted direction of bias and whether bias threatens the conclusions. No arterial-pressure threshold will be proposed for clinical use unless these sources of evidence are sufficiently concordant.

### Protocol amendments

The protocol specifies the management-strategy eligibility rules, troponin ascertainment groups, confounding and model-selection criteria, and ROBINS-E threshold for the primary synthesis before definitive searches begin. Further important deviations from the registered protocol will be dated, justified, recorded on OSF, reflected in PROSPERO when applicable and reported in the final review.

### Patient and public involvement

Patients and the public were not involved in developing this protocol and are not planned to participate in review conduct or reporting.

### Ethics and dissemination

This systematic review will use published or publicly available aggregate data and will not recruit participants, access identifiable private information or alter patient care; ethical approval and individual consent are therefore not required. Findings will be submitted to a peer-reviewed journal, presented at scientific meetings and communicated through the OSF project. On completion of the review, the search strategies, amendment history, aggregate extraction dataset and analysis code will be made publicly available through the OSF project at https://osf.io/rb6fn/.

## Discussion

The four analytic domains separate observational exposure associations from comparative management effects and keep AKI distinct from myocardial injury. An association between lower arterial pressure and injury does not establish that increasing pressure will improve outcomes. Effects of multicomponent management strategies will be attributed to the strategy as a whole unless the arterial-pressure component can be isolated. Outcome surveillance, alternative exposure definitions and overlapping cohorts will be considered when judging whether studies are comparable.

Interpretation may be limited by small study numbers, non-standard hypotension metrics, residual confounding and incomplete creatinine or troponin surveillance. A postoperative troponin elevation without baseline or serial measurements may reflect chronic rather than acute injury. Blood loss, transfusion, fluid administration and vasoactive treatment may precede hypotension or occur in response to it. Cumulative intraoperative measurements may therefore make it difficult to distinguish confounders from downstream variables and assess whether adjustment was appropriate. These limitations will be considered alongside baseline renal function, cardiovascular disease, cirrhosis and resection extent when judging certainty.

## Supporting information

S1 Checklist PRISMA-P 2015 checklist. (DOCX)

S1 File Search strategy and PRESS peer-review methods. (DOCX)
