# Supplementary material for: Intraoperative hypotension and postoperative acute kidney injury or myocardial injury after liver resection: A protocol for a systematic review and meta-analysis
Source: PLoS One. 2026 Oct 12;21(10):e0357750. doi: 10.1371/journal.pone.0357750 (PMC13657278; doi:10.1371/journal.pone.0357750)
Supplement: Supplementary file 1 [file pone.0357750.s001.docx]

# PRISMA-P (Preferred Reporting Items for Systematic review and Meta-Analysis Protocols) 2015 Checklist

Recommended items to address in a systematic review protocol*

| Section & Topic | Item No. | Checklist Item | **Reported in section or table** |
| --- | --- | --- | --- |
| ADMINISTRATIVE INFO |  |  |  |
| Title |  |  |  |
| Identification | 1a | Identify the report as a protocol of a systematic review | Title page |
| Update | 1b | If the protocol is for an update of a previous systematic review, identify as such | Not applicable (new review) |
| Registration |  |  |  |
| Registration | 2 | If registered, provide the name of the registry (such as PROSPERO) and registration number | Title page; Abstract; Design, reporting and registration |
| Authors |  |  |  |
| Contact | 3a | Provide name, institutional affiliation, e-mail address of all protocol authors; provide physical mailing address of corresponding author | Title page |
| Contributions | 3b | Describe contributions of protocol authors and identify the guarantor of the review | Submission form: Author Contributions |
| Amendments |  |  |  |
| Amendments | 4 | If the protocol represents an amendment of a previously completed or published protocol, identify as such and list changes; otherwise, state plan for documenting important protocol amendments | Design, reporting and registration; Protocol amendments |
| Support |  |  |  |
| Sources | 5a | Indicate sources of financial or other support for the review | Submission form: Financial Disclosure |
| Sponsor | 5b | Provide name for the review funder and/or sponsor | Not applicable: no funder or sponsor (Financial Disclosure) |
| Role of Sponsor or Funder | 5c | Describe roles of funder(s), sponsor(s), and/or institution(s), if any, in developing the protocol | Not applicable: no funder or sponsor (Financial Disclosure) |
| INTRODUCTION |  |  |  |
| Rationale |  |  |  |
| Rationale | 6 | Describe the rationale for the review in the context of what is already known | Introduction |
| Objectives |  |  |  |
| Objectives | 7 | Provide an explicit statement of the question(s) the review will address with reference to participants, interventions, comparators, and outcomes (PICO) | Introduction; Review questions and analytic domains; Table 1 |
| METHODS |  |  |  |
| Eligibility Criteria |  |  |  |
| Eligibility criteria | 8 | Specify the study characteristics (such as PICO, study design, setting, time frame) and report characteristics (such as years considered, language, publication status) to be used as criteria for eligibility for the review | Eligibility criteria; Tables 1-2; Participants and setting; Exposure and intervention definitions; Outcomes; Study designs and report characteristics |
| Information Sources |  |  |  |
| Information sources | 9 | Describe all intended information sources (such as electronic databases, contact with study authors, trial registers or other grey literature sources) with planned dates of coverage | Design, reporting and registration; Information sources and search strategy |
| Search Strategy |  |  |  |
| Search strategy | 10 | Present draft of search strategy to be used for at least one electronic database, including planned limits, such that it could be repeated | Information sources and search strategy; S2 File: PubMed - draft strategy |
| Study Records |  |  |  |
| Data management | 11a | Describe the mechanism(s) that will be used to manage records and data throughout the review | Study records and selection; Data collection and management |
| Selection process | 11b | State the process that will be used for selecting studies (such as two independent reviewers) through each phase of the review (that is, screening, eligibility and inclusion in meta-analysis) | Study records and selection |
| Data collection process | 11c | Describe planned method of extracting data from reports (such as piloting forms, done independently, in duplicate), any processes for obtaining and confirming data from investigators | Data collection and management |
| Data Items |  |  |  |
| Data items | 12 | List and define all variables for which data will be sought (such as PICO items, funding sources), any pre-planned data assumptions and simplifications | Data items and multiplicity; Confounding and model selection |
| Outcomes and Prioritization |  |  |  |
| Outcomes and prioritization | 13 | List and define all outcomes for which data will be sought, including prioritization of main and additional outcomes, with rationale | Outcomes; Data items and multiplicity; Table 3 |
| Risk of Bias |  |  |  |
| Risk of bias in individual studies | 14 | Describe anticipated methods for assessing risk of bias of individual studies, including whether this will be done at the outcome or study level, or both; state how this information will be used in data synthesis | Risk-of-bias assessment; Data synthesis; Sensitivity analyses |
| Data Synthesis |  |  |  |
| Data synthesis | 15a | Describe criteria under which study data will be quantitatively synthesised | Data synthesis; Table 3 |
|  | 15b | If data are appropriate for quantitative synthesis, describe planned summary measures, methods of handling data and methods of combining data from studies, including any planned exploration of consistency (such as I^2^, Kendall’s τ) | Effect measures; Data synthesis; Heterogeneity and additional analyses |
|  | 15c | Describe any proposed additional analyses (such as sensitivity or subgroup analyses, meta-regression) | Heterogeneity and additional analyses; Sensitivity analyses |
|  | 15d | If quantitative synthesis is not appropriate, describe the type of summary planned | Data synthesis (synthesis without meta-analysis) |
| Meta-bias(es) |  |  |  |
| Meta-bias(es) | 16 | Specify any planned assessment of meta-bias(es) (such as publication bias across studies, selective reporting within studies) | Reporting biases |
| Confidence in Evidence |  |  |  |
| Confidence in cumulative evidence | 17 | Describe how the strength of the body of evidence will be assessed (such as GRADE) | Certainty of evidence and interpretation |

*It is strongly recommended that this checklist be read in conjunction with the PRISMA-P Explanation and Elaboration for important clarification on the items. Amendments to a review protocol should be tracked and dated. The copyright for PRISMA-P (including checklist) is held by the PRISMA-P Group and is distributed under a Creative Commons Attribution Licence 4.0.

From: *Shamseer L, Moher D, Clarke M, Ghersi D, Liberati A, Petticrew M, Shekelle P, Stewart LA; PRISMA-P Group. Preferred reporting items for systematic review and meta-analysis protocols (PRISMA-P) 2015: elaboration and explanation. BMJ. 2015;350:g7647. doi:10.1136/bmj.g7647. Published correction: BMJ. 2016;354:i4086. doi:10.1136/bmj.i4086.*
