# Supplementary material for: Intraoperative hypotension and postoperative acute kidney injury or myocardial injury after liver resection: A protocol for a systematic review and meta-analysis
Source: PLoS One. 2026 Oct 12;21(10):e0357750. doi: 10.1371/journal.pone.0357750 (PMC13657278; doi:10.1371/journal.pone.0357750)
Supplement: Supplementary file 2 [file pone.0357750.s002.docx]

**S2 FILE**

**Search strategy and PRESS peer-review methods**

# SEARCH STRATEGY

## Review title

Intraoperative hypotension and postoperative acute kidney injury or myocardial injury after liver resection: a protocol for a systematic review and meta-analysis

## Protocol and registrations

Version 1.0; 13 August 2026

PROSPERO: CRD420261476933

OSF registration DOI: https://doi.org/10.17605/OSF.IO/3GCY2

## Search development and peer review

Yi Duan developed the draft PubMed strategy. Before the definitive search, Meng Li, who did not participate in its development, will independently peer-review it using the PRESS 2015 evidence-based checklist. Yi Duan will address the comments and revise the strategy where appropriate. Meng Li will then translate the approved strategy for Embase.com, Web of Science Core Collection and CENTRAL. No language, date, adult or study-design filter will be applied electronically. Definitive searches are planned for 28 September 2026.

# PubMed - draft strategy

**#1 Liver-resection population**

"Hepatectomy"[Mesh]

OR hepatectom*[tiab]

OR "liver resection"[tiab]

OR "liver resections"[tiab]

OR "hepatic resection"[tiab]

OR "hepatic resections"[tiab]

OR "liver surgery"[tiab]

OR "hepatic surgery"[tiab]

OR "living donor hepatectomy"[tiab]

OR "living-donor hepatectomy"[tiab]

**#2 Arterial pressure and haemodynamic concepts**

"Hypotension"[Mesh]

OR "Blood Pressure"[Mesh]

OR "Hemodynamics"[Mesh]

OR "Intraoperative Care"[Mesh]

OR hypotens*[tiab]

OR "mean arterial pressure"[tiab]

OR "mean arterial pressures"[tiab]

OR "arterial pressure"[tiab]

OR "arterial pressures"[tiab]

OR "blood pressure"[tiab]

OR "blood pressures"[tiab]

OR "intraoperative MAP"[tiab]

OR "time weighted average"[tiab]

OR "time-weighted average"[tiab]

OR "area under the threshold"[tiab]

OR "area under threshold"[tiab]

OR hemodynamic*[tiab]

OR haemodynamic*[tiab]

OR "goal directed"[tiab]

OR "goal-directed"[tiab]

OR vasopressor*[tiab]

OR norepinephrine[tiab]

OR noradrenaline[tiab]

OR phenylephrine[tiab]

**#3 Broad haemodynamic path**

#1 AND #2

**#4 Kidney and myocardial injury concepts**

"Acute Kidney Injury"[Mesh]

OR "Myocardial Infarction"[Mesh]

OR "Troponin"[Mesh]

OR "acute kidney injury"[tiab]

OR "acute kidney injuries"[tiab]

OR "acute renal injury"[tiab]

OR "acute renal failure"[tiab]

OR "kidney dysfunction"[tiab]

OR "renal dysfunction"[tiab]

OR "renal failure"[tiab]

OR creatinine[tiab]

OR dialysis[tiab]

OR "renal replacement therapy"[tiab]

OR "myocardial injury"[tiab]

OR "myocardial injuries"[tiab]

OR "perioperative myocardial injury"[tiab]

OR "peri-operative myocardial injury"[tiab]

OR "myocardial injury after noncardiac surgery"[tiab]

OR "myocardial injury after non-cardiac surgery"[tiab]

OR MINS[tiab]

OR "myocardial infarction"[tiab]

OR troponin*[tiab]

**#5 Organ-injury path**

#1 AND #4

**#6 Final PubMed set**

#3 OR #5

## Other bibliographic databases

**•** The Embase.com strategy will use Emtree and title, abstract and keyword fields while retaining the two search paths.

**•** The Web of Science Core Collection strategy will use Topic fields and database-supported proximity and truncation operators.

**•** The CENTRAL strategy will use MeSH and free-text terms while retaining the two search paths.

**•** Each bibliographic database will be searched from inception.

## Trial registers

ClinicalTrials.gov and WHO ICTRP will be searched using combinations of hepatectomy, liver resection, hepatic resection and living donor hepatectomy, without an outcome restriction. Each record will be screened for an eligible arterial-pressure exposure or comparative haemodynamic strategy and an eligible outcome.

## Supplementary searching

We will conduct backward and forward citation searches of included studies and relevant reviews, examine PubMed related articles, contact authors when required and consult Scite for citation context and notices of correction or retraction.

## Search reporting

For each source, the final review will report the database or register, platform, complete strategy, search date, coverage dates, result count before deduplication and peer-review status.

## Update rule

If final analysis occurs more than six months after the initial search, all bibliographic searches will be updated.
